# First Molecular Characterization of Feline Immunodeficiency Virus in Domestic Cats from Mainland China

**DOI:** 10.1371/journal.pone.0169739

**Published:** 2017-01-20

**Authors:** Jilei Zhang, Liang Wang, Jing Li, Patrick Kelly, Stuart Price, Chengming Wang

**Affiliations:** 1 Jiangsu Co-Innovation Center for the Prevention and Control of Important Animal Infectious Diseases and Zoonoses, Yangzhou University College of Veterinary Medicine, Yangzhou, Jiangsu, China; 2 Department of Clinical Sciences, Ross University School of Veterinary Medicine, Basseterre, Saint Kitts & Nevis, West Indies; 3 Department of Pathobiology, College of Veterinary Medicine, Auburn, Alabama, United States of America; CEA, FRANCE

## Abstract

The feline immunodeficiency virus (FIV) is a retrovirus of the *Lentivirus* genus that was initially isolated from a colony of domestic cats in California in 1986 and has now been recognized as a common feline pathogen worldwide. To date, there is only one recent serology-based report on FIV in mainland China which was published in 2016. We designed this study to investigate the molecular prevalence and diversity of feline immunodeficiency virus (FIV) in domestic cats from mainland China. We studied the prevalence of FIV in whole blood samples of 615 domestic cats in five cities (Beijing, Guangzhou, Nanjing, Shanghai and Yangzhou) of mainland China and examined them using FRET-PCR (Fluorescence Resonance Energy Transfer-Polymerase Chain Reaction) and regular PCRs for the *gag* and *env* genes. Overall, 1.3% (8/615) of the cats were positive for provirus DNA with nucleotide analysis using PCRs for the *gag* and *env* sequences showing the cats were infected with FIV subtype A. This is the first molecular characterization of FIV in mainland China and the first description of subtype A in continental Asia.

## Introduction

The feline immunodeficiency virus (FIV) is a retrovirus of the *Lentivirus* genus that was initially isolated from colony of domestic cats in California in 1986 and has now been recognized as a common feline pathogen worldwide [1–4]. Infected cats may be asymptomatic for many years during which there is progressive disruption of immune function which might lead to a terminal phase with various clinical infections that is referred to as the feline acquired immunodeficiency syndrome [5]. Transmission of FIV is principally by parenteral inoculation of the virus in blood and saliva, presumably during fighting. Male cats are more commonly infected than females and overall prevalence rates in cats vary geographically, mostly from around 2% to 30% [5].

FIV occurs as seven subtypes or clades (A, B, C, D, E, F and U-NZenv) based on nucleotide sequence diversity of the envelope (*env*) gene [6–9]. The distribution of the clades varies with subtypes A and B being most common, and occurring very widely [8, 10]. Subtype A is common in Australia, New Zealand, the western part of the United States, South Africa and northwestern Europe [8]. Subtype C has been identified in Europe, Africa, Southeast Asia, New Zealand and Canada while subtypes D and E are found only infrequently, originally in Japan, Canada and Argentina [11–14]. Subtype F has only been described from Portugal and the US and the U-NZenv subtype only from New Zealand [7, 9, 15]. There is only limited data on the genotypes of the latter two subtypes.

To date, there have been five FIV-related reports in Taiwan [16–20], but only little data on FIV in mainland China. A study on wild Pallas’ cats from China and other Asian countries identified a unique monophyletic lineage of the FIV most closely related to FIV of African wild cats [21–22].

In the only work on domestic cats, a serosurvey using a commercial test kit (SNAP^®^ Feline Triple^®^ Test, IDEXX Laboratories, Westbrook, ME, USA) found 9% (33/362) of cats studied in Lanzhou, northwestern China, were positive [23]. To provide further information on FIV infections we carried out a molecular survey on cats from five areas in mainland China.

## Materials and Methods

The study was reviewed and approved by the Institutional Animal Care and Use Committee of the Yangzhou University College of Veterinary Medicine. Between April 2013 and June 2015, whole blood samples were collected from 615 cats in five cities (Beijing, Guangzhou, Nanjing, Shanghai and Yangzhou) in four provinces of mainland China. The cats from Yangzhou were apparently healthy animals in a shelter while those from the other cities were cats presenting to veterinary clinics for routine health examinations and vaccinations and neutering or with a variety of conditions including fever, stomatitis, and renal failure. All blood samples were collected into EDTA-containing tubes and stored at -80°C until DNA extraction.

DNA was extracted from whole blood samples with the QIAamp^®^ DNA Blood Mini Kit (QIAgen, Valencia, USA) following the protocol of the manufacturer. A negative control, diethylpyrocarbonate (DEPC)-treated ddH_2_O, was used for extraction after every 24 blood samples to confirm the absence of carry-over contamination during DNA extraction.

The FIV FRET-PCR was performed in a LightCycler 480-II real-time PCR platform as described previously [24]. This PCR method can detect single copies of a 176-bp *gag* gene fragment of the FIV provirus genome and can be used to differentiate subtypes A to E [24]. Positive controls consisted of nucleotide fragments of the *gag* regions of FIV subtypes A, B1, B2/E, C and D that were prepared as described previously [24]. Products obtained in the FIV FRET-PCR were further verified by electrophoresis through 2% agarose gels (BIOWEST^®^, Hong Kong, China), purified with the QIAquick PCR Purification Kit (Qiagen, Germany), and sequenced with forward and reverse primers (BGI Shanghai, China).

**Fig 1:**
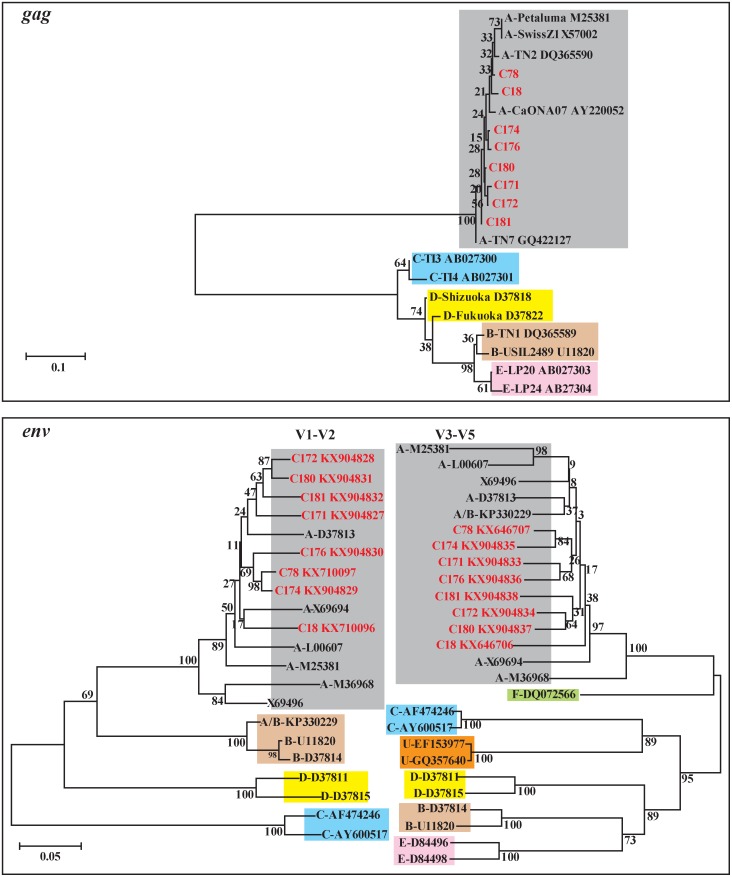
Phylogeny of *gag* and *env* genes of FIV. *Gag* sequences (176-bp) of FIV strains identified in this study and representatives of the five subtypes with sequences in GenBank. In addition, a 374-bp region encompassing V1 to V2 is shown on the left of the bottom panel, and a 502-bp region encompassing V3 to V5 on the right panel. The *env* sequences of the FIV strains identified in this study (in red) are compared with the sequences of representatives of the FIV subtypes with sequences in GenBank; five for V1 to V2 and seven for V3 to V5. Branch lengths are measured in nucleotide substitutions and numbers show branching percentages in bootstrap replicates. Scale bar represents the percent sequence diversity.

The *env* sequences of eight FIV subtypes (sybtype A: M25381, L00607, X69496, D37813, X69694, M36968; subtype A/B: KP330229; subtype B: D37814, U11820; subtype C: AF474246, AY600517; subtype D: D37811, D37815; subtype E: D84496, D84498; subtype F: DQ072566; subtype U: EF153977, GQ357640) (Fig 1) were obtained from GenBank (www.ncbi.nlm.nih.gov/). The Clustal Multiple Alignment Algorithm was used on the V1-V2 and V3-V4 regions common to the *env* of all the above FIV subtypes to identify polymorphic regions that would enable us to differentiate between subtypes. The primers to amplify the polymorphic regions were synthesized by GenScript (GenScript, Nanjing, China). Standard PCRs were performed with the primers we designed against a 374-bp segment in the V1-V2 region (forward: GAAGAAGGAAATGCAGGTAAGTTTAGAA; reverse: GGTGCCCAACAATCCCAAAA) and a 680-bp segment of V3-V5 (forward: ATACCAAAATGTGGATGGTGGAA; reverse: TAATCCTGCTACTGGGTTATACCAATT). The primers for the V1-V2 region (first segment of the *env*) amplify subtypes A to E while those for the V3-V5 region (second segment of *env*) detect all subtypes (A to F and U-NZenv). Positive controls consisted of FIV subtypes A, B and C identified in a previous study [24]. The standard PCRs were performed in a Roche LightCycler II PCR platform. Each reaction was performed with a 20μl final volume containing 10μl of extracted nucleotides, 1×PCR buffer, 1μM forward primer, 1μM reverse primer, 2 unit Taq DNA polymerase and 200μM dNTP. Thermal cycling consisted of 18 high-stringency step-down cycles followed by 30 relaxed-stringency cycles. The cycling parameters for PCR were 6 × 1 sec at 95°C, 12 sec at 72°C, 30 sec at 72°C; 9 × 1 sec at 95°C, 12 sec at 70°C, 30 sec at 72°C; 3 × 1 sec at 95°C, 12 sec at 68°C, 30 sec at 72°C; 30 × 1 sec at 95°C, 8 sec at 56°C, 30 sec at 67°C, 30 sec at 72°C. Products were verified by gel electrophoresis and sequenced with forward and reverse primers using the Sanger method (BGI, Shanghai, China).

The *gag* and *env* sequences we obtained were aligned with similar sequences in GenBank with the Clustalx 1.83 alignment software. Phylogenetic trees were constructed by the neighbor-joining method using the Kimura 2-parameter model with MEGA 6.0. Bootstrap values calculated using 500 replicates.

## Results

We analyzed blood samples from 615 cats from Beijing (n = 138), Guangzhou (75), Nanjing (146), Shanghai (143) and Yangzhou (113). Background data was available for 514 cats of which 383 were owned and kept mainly indoors and 131 were strays; 278 were male and 236 were female. Estimated age data was available for 458 cats which were placed into one of the following arbitrary age groups: 68 kittens (< 6 m), 225 young adults (6 m to 4 yrs), 101 adults (4 to 10yrs) and 64 older cats (>10yrs).

The FRET-PCR followed by confirmatory sequencing showed that 1.3% (8/615) of the cats were positive for FIV. All the FIV-positive cats were male cats from Guangzhou (n = 1), Shanghai (3) and Nanjing (4) (Table 1). Seven of these 8 FIV-positive cats were sick with clinical signs such as stomatitis, salivation and anorexia. The melting point and the *gag* sequence analyses of the FRET-PCR showed all the positive sequences belonged to FIV subtype A. They had 97%-99% (2-5/164 nucleotide mismatches) similarity with the FIV subtype A TN7 strain (GQ422127) from Canada, and 97%-98% (2-4/164 mismatches) similarity with a FIV subtype A CaONA07 strain (AY225009) from Canada.

**Table 1:** FIV-positive cats identified in this study.

Cat	City	Age (year)	Gender	Source	Health status
C18	Guangzhou	1.0	Neutered male	Domestic cat	Renal failure
C180	Nanjing	3.0	Intact male	Feral cat	Stomatitis
C181	Nanjing	3.0	Intact male	Feral cat before adoption	Depression
C171	Nanjing	1.5	Intact male	Feral cat before adoption	Stomatitis
C172	Nanjing	3.0	Intact male	Domestic cat	Stomatitis
C174	Shanghai	0.25	Intact male	Domestic cat	Fever, 41.3°C
C176	Shanghai	3.0	Neutered male	Domestic cat	Feline calicivirus infection
C78	Shanghai	10.0	Intact male	Domestic cat	Apparently healthy

The sequences of the V1-V2 *env* region (GenBank accession number: KX710096- KX710097 and KX904827-KX904832) in the positive cats were all similar (90%-97% identity) with six being most closely related to the UK2 strain. This is a FIV subtype A from Scotland (X69494) which has 91% similarity with C18, C172, C176 and C180 (32–34 mismatches) and 93% similarity with C78 and C174 (26 and 28 mismatches, respectively) [25]. In the remaining two positive cats, one (C171) had a strain most closely related to the Sendai1 strain, a FIV subtype A from Japan (D37814) (91% similarity, 32/374), and the other (C181) a strain with 91% similarity to UK2 strain and Sendai1 strain (38/374). (Table 2) [26].

**Table 2:** Percent similarities (upper-right diagonal half) and actual numbers of mismatches (lower-left diagonal half) in the *env* V1-V2 sequences (374bp) of two FIV positive cats from China and representatives of the four FIV subtypes with sequences on GenBank.

	C18	C78	C171	C172	C174	C176	C180	C181	UK2	Sendai1	UK8	Dixon	Petaluma	PPR	FDS	Sendai2	USIL	C	C36	Shizuoka	Fukuoka
C18^a^		93	90	93	93	90	93	92	**91**	90	88	90	89	85	76	76	73	65	65	70	70
C78	27		92	94	98	94	94	94	**93**	92	89	92	92	85	76	75	72	66	65	71	70
C171	36	28		93	93	90	94	92	90	**91**	87	89	88	84	75	73	71	66	66	70	70
C172	28	24	26		94	91	97	94	**91**	91	88	91	89	84	77	75	72	66	66	72	71
C174	28	7	25	22		94	95	94	**93**	92	89	92	92	85	76	75	72	66	65	71	70
C176	38	22	40	34	22		91	91	**91**	90	88	90	89	84	77	76	73	66	65	71	69
C180	28	22	24	12	19	34		95	**91**	91	88	91	90	84	76	74	71	66	66	72	71
C181	31	28	32	24	26	38	21		**91**	**91**	87	90	89	84	75	74	72	67	66	71	70
A-UK2	**33**	**28**	37	**32**	**26**	**34**	**32**	**38**		90	88	91	90	83	75	74	71	66	64	70	69
A-Sendai1	38	29	**32**	34	28	38	34	**38**	37		88	91	90	84	76	74	72	66	66	69	67
A-UK8	43	41	37	43	40	46	46	53	44	44		88	87	90	76	75	72	66	66	69	68
A-Dixon	38	31	43	35	31	37	34	41	34	36	44		91	84	76	74	72	65	64	70	67
A-Petaluma	42	31	46	40	31	43	38	46	37	37	49	34		85	77	76	74	66	66	72	70
A-PPR	56	55	62	61	56	61	59	63	65	60	40	60	57		76	75	72	66	65	69	67
A/B-FDS	91	91	95	89	90	87	93	98	96	92	90	92	89	91		95	94	68	68	70	71
B-Sendai2	94	97	102	96	96	93	101	104	100	99	96	99	94	96	19		97	67	67	70	70
B-USIL2489	103	106	113	107	107	104	111	112	109	108	107	106	102	105	24	12		66	65	68	69
C-C	133	131	131	129	133	133	130	130	133	129	131	136	128	131	126	129	134		95	63	64
C-C36	133	134	132	130	136	136	130	133	137	132	131	140	130	134	124	130	137	20		64	65
D-Shizuoka	114	111	114	107	111	113	107	115	114	119	105	115	105	120	111	114	119	143	143		92
D-Fukuoka	116	114	116	110	116	117	110	118	119	125	120	125	115	125	110	113	118	140	139	32	

^a^ The GenBank Accession numbers of the China strains are C18(KX710096); C78 (KX710097), C171 (KX904827), C172 (KX904828), C174 (KX904829), C176 (KX904830), C180 (KX904831) and C181 (KX904832), while those of previously reported FIV are: subtype A, UK2 (X69494), Sendai1 (D37813), UK8 (X69496), Dixon (L00607), Petaluma (M25381), PPR (M36968); subtype A/B, FDSydneyC36 (KP330229); subtype B, Sendai2 (D37814), USIL2489 (U11820); subtype C, C (AF474246), C36 (AY600517); subtype D, Shizuoka (D37811), Fukuoka (D37815).

The sequences of the *env* V3-V5 segment amplicons of the eight positive cats (GenBank accession number: KX646706-KX646707 and KX904833-KX904838) differed by 3%-7% (27–53 mismatches) (Table 3). Five were most closely related to the UK2 strain, a FIV subtype A from Scotland (X69496), with 94–95% similarity (38–43 mismatches) to C171, C172, C174, C176 and C180 (Table 3) [19]. The other three positivities were most closely related to FIV subtype A/B strain FDSydneyC36 from Australia (KP330229) which had 94% (636/677) identity with C18, 96% (652/683) identity with C78 and 95% (638/680) identity with C181, respectively (Table 3, Fig 1) [26].

**Table 3:** Percent similarities (upper-right diagonal half) and actual numbers of mismatches (lower-left diagonal half) in the *env* V3-V5 sequences (C18:677bp and C78:683bp) of two FIV positive cats from China and representatives of each of the seven FIV subtypes with sequences on GenBank.

	C18	C78	C171	C172	C174	C176	C180	C181	UK8	Sendai1	UK2	Dixon	PPR	Petaluma	FDS	Sendai2	USIL	C	C36	Shizuoka	Fukuoka	LP3	LP20
C18^a^		94	93	94	93	94	94	95	93	93	93	91	90	90	**94**	81	80	79	79	79	80	82	82
C78	45		95	95	97	96	94	94	95	94	93	93	90	92	**96**	80	80	80	79	80	81	81	81
C171	53	40		95	94	95	94	94	**94**	94	93	92	91	90	94	81	80	79	80	79	80	80	80
C172	44	38	38		96	95	96	96	**95**	94	93	94	91	92	95	81	79	80	80	79	80	81	81
C174	53	27	40	34		95	94	95	**94**	94	92	92	90	91	95	80	78	78	78	78	80	80	80
C176	51	34	36	36	34		94	94	**94**	94	93	92	91	91	94	81	80	80	80	80	81	81	81
C180	51	47	42	32	42	42		95	**94**	93	93	93	90	91	94	80	79	80	80	80	80	80	80
C181	48	47	45	34	38	42	33		94	93	93	92	90	92	**95**	80	79	79	79	80	81	81	81
UK8	56	42	**40**	**38**	**38**	**42**	**43**	49		95	94	93	90	92	95	80	79	79	80	79	81	81	81
A-Sendai1	50	43	49	46	49	51	59	55	44		93	93	90	92	96	80	79	80	80	79	80	80	80
A-UK2	56	49	49	51	53	51	51	49	44	55		92	90	90	94	80	79	80	80	79	81	81	81
A-Dixon	66	49	55	49	56	55	49	55	47	54	59		89	92	94	81	80	80	79	81	81	81	80
A-PPR	68	70	70	69	79	72	80	78	77	72	72	80		88	91	82	82	82	82	80	81	81	81
A-Petaluma	73	55	70	65	67	62	67	63	58	65	69	56	91		92	81	79	78	79	80	81	81	80
A/B-FDS	**41**	**31**	45	40	42	45	46	**42**	40	32	45	50	70	60		81	80	80	80	80	81	81	81
B-Sendai2	143	147	140	142	148	140	141	142	144	159	138	139	137	141	141		95	81	80	82	86	85	85
B-USIL2489	145	147	144	148	152	141	144	144	150	152	154	146	136	149	145	41		80	80	83	87	84	85
C-C	156	154	150	148	157	148	144	152	150	147	153	156	142	159	150	138	137		99	80	80	80	80
C-C36	153	153	146	146	154	144	144	148	145	146	150	154	139	156	147	139	135	13		80	80	80	81
D-Shizuoka	160	147	150	154	159	146	152	152	152	153	150	150	148	142	146	131	126	151	148		92	82	82
D-Fukuoka	144	138	139	140	141	133	136	133	135	145	143	141	139	140	139	102	87	139	137	70		85	84
E-LP3	138	141	139	140	139	135	142	133	135	144	146	141	142	139	138	106	109	143	136	137	108		95
E-LP20	136	141	139	138	141	133	140	135	139	147	144	143	140	143	139	104	104	139	133	139	115	35	

^a^ The GenBank Accession numbers of the China strains are C18 (KX646706), C78 (KX646707), C171 (KX904833), C172 (KX904834), C174 (KX904835), C176 (KX904836), C180 (KX904837) and C181 (KX904838), while those of previously reported FIV are: subtype A, UK8 (X69496), Sendai1 (D37813), UK2 (X69494), Dixon (L00607), PPR (M36968), Petaluma (M25381); subtype A/B, FDSydneyC36 (KP330229); subtype B, Sendai2 (D37814), USIL2489 (U11820); subtype C, C (AF474246), C36 (AY600517); subtype D, Shizuoka (D37811), Fukuoka (D37815); subtype E, LP3 (D84496), LP20 (D84498).

**Fig 2:**
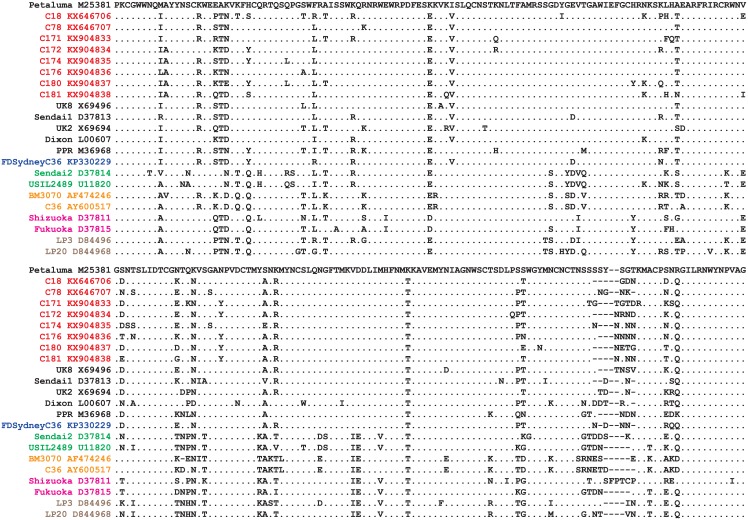
Alignment of amino acid of FIV on envelop protein V3-V5 regions. V3-V5 region amino acid sequences of FIV strains identified in this study (red font) were aligned with those of representatives FIV subtypes in GenBank (black for subtype A, blue for subtype A/B; green for subtype B; orange for subtype C, pink for subtype D, and brown for subtype E). Identical nucleotides at given positions are represented by dots (·), gaps are represented by dashes (-).

The phylogenetic trees we generated (Fig 1) that were based on the nucleotide sequences of our mainland China FIV strains and representative strains of FIV from GenBank clearly demonstrated that our Chinese strains were members of subtype A. In addition, the V3-V5 amino acid sequences of the envelop protein for FIV cats in this study were aligned with those of representative strains of FIV from GenBank (Fig 2).

## Discussion

The results of our study confirm the presence of the FIV in mainland China and add to the known distribution range of the virus in the country. We found a low prevalence but the cats we studied were predominantly indoor pets that had little contact with other cats. Elsewhere, such cats also have a low prevalence of infection, for example 0.7% in the USA [24]. Why we found no infected cats in the shelter population from Yangzhou is unclear, as feral cats often have a high prevalence of FIV infection, for example 18% in the US [24].

Previous studies have shown cats infected with FIV do not have decreased longevity [27] and that it is only after relatively prolonged infection that immunosuppression occurs and clinical signs become apparent [28]. It was unexpected, then, that seven of the cats we found positive for FIV clinically ill although still relatively young (3 years of age or younger). Unfortunately, there was little or no laboratory data available on these cats and we were not able to establish what, if any, role the FIV infections might have played in the clinical signs that were reported.

Previous studies have shown that PCRs for FIV provirus detection can have a wide range of sensitivities (41–93%) [29]. This relatively poor sensitivity might be as a result of the very low levels of provirus that can be present in infected cats, particularly in apparently healthy animals, but can also be due to variability in the proviral genome of the FIV; there can be up to 26% polymorphism between serotypes in the *env* and *gag* [30, 31]. Further, recombination with sometimes complex patterns resulting from co-infections or super-infections is also not uncommon in the FIVs [9, 32]. Because of the wide range of subtypes of FIVs and their high evolutionary rate, it is difficult to develop a PCR that is generic enough to amplify all subtypes and yet maintain high sensitivity [33]. The FRET-PCR we used against the *gag* has been shown to be sensitive, detecting single copies of the target, and capable of differentiating FIV subtypes A, B, C, D and E [24]. Similarly, the primers we developed against the V1-V2 region of the first segment of the *env* gene amplified subtypes A to E and enabled their differentiation with sequencing. We could not establish if our primers amplified subtype F and subtype U-NZenv as there are no sequence data for this region on GenBank for these two serotypes. There is sequence data, however, for the V3-V5 region of all the FIV subtypes and the primers we developed for this second segment of the *env* were capable of detecting all subtypes, that is A to F and also U-NZenv. The PCRs we performed in our study thus enabled us to detect low copy numbers of FIV and also to detect all the recognized subtypes.

In our study, all the FIV positive isolates we detected belonged to subtype A which occurs widely around the world with most isolates being from Australia, New Zealand, North America, South Africa and Europe [10, 34]. Isolates from countries closer to mainland China have included subtypes A, B, C and D from Japan [14], subtype C from Korea and Vietnam [13, 35], and subtype D from Thailand [36]. Our description of subtype A in mainland China is thus the first description of this subtype in the country and, to the best of our knowledge, on the mainland of Asia.

Of note is our finding that the sequences of the second segment of the *env* in three of our mainland China FIV strains (C18, C78 and C181) were very similar to the FDSydneyC36 (41, 31 and 42 mismatches, respectively) (Table 3, Fig 2). The sequences of the first segment of the *env*, however, were relatively distant (91, 91 and 98 mismatches, respectively), being more closely aligned with representatives of the FIV subtype B (Table 2, Fig 2). This difference is explained by the fact that the FDSydneyC36 strain, from a cat immunized with a commercial FIV vaccine [26], is a recombinant strain of FIV, subtype A/B. The second segment of the *env* is assigned to subtype A while the first segment is assigned to subtype B.

Our findings of FIV subtype A and the serological evidence of infections presented by Cong et al. [23] should alert Chinese veterinarians to the possibility of infections in their feline patients. Although clinical signs resulting from FIV infection are highly variable and unpredictable, cats infected with subtype A have been found to remain asymptomatic for longer and have lower viral loads than cats infected with subtype C [34, 37, 38]. The subtype A FIV strains are often neurotrophic and can produce neurological signs, most commonly behavioral changes but also seizures, paresis, multifocal motor abnormalities, impaired learning and disrupted sleep patterns [5]. Currently there is only one registered FIV vaccine which is composed of two FIV subtypes, A and D. The vaccine is reported to confer protection against subtypes A, B and D and might then be useful in mainland China where [32, 39], to the best of our knowledge, vaccination is seldom if ever performed. A recent study, however, has shown the vaccine does not confer solid protection and breakthroughs were found with FIV subtypes A, F, A/F and D/F [40]. Further studies on the usefulness of vaccination under conditions of natural challenge are required, particularly in Asian countries where subtype C is prevalent.

In conclusion, our study has shown that FIV subtype A occurs in mainland China and continental Asia. Larger studies are indicated to further determine the subtypes present in the region which will facilitate the development of accurate diagnostic tools and control programs.
